# A novel terpolymer nanocomposite (carboxymethyl β-cyclodextrin–nano chitosan–glutaraldehyde) for the potential removal of a textile dye acid red 37 from water

**DOI:** 10.3389/fchem.2023.1115377

**Published:** 2023-02-01

**Authors:** Hemmat A. Elbadawy, Ali El-Dissouky, Seham M. Hussein, Sara R. El-Kewaey, Souad A. Elfeky, Gamal El-Ghannam

**Affiliations:** ^1^ Chemistry Department, Faculty of Science, Alexandria University, Alexandria, Egypt; ^2^ Department of Laser Applications in Metrology, Photochemistry, and Agriculture, National Institute of Laser Enhanced Sciences (NILES), Cairo University, Cairo, Egypt

**Keywords:** terpolymer, CM-βCD:nChi:Glu, acid red 37, textile dye, adsorption capacity, % removal

## Abstract

Carboxymethyl β-cyclodextrin–nanochitosan–glutaraldehyde (CM-βCD:nChi:Glu) terpolymer was prepared as a nano-adsorbent for the removal of the anionic textile dye, acid red 37. The terpolymer nanocomposite formation and characterization were clarified by FTIR, XRD, scanning electron microscopy, TEM, Brunauer–Emmett–Teller specific surface area (BET-SSA), and zeta potential. The removal of the textile dye was investigated by using the batch adsorption method, investigating the effect of pH, dye concentration, adsorbent dose, contact time, and temperature. The results revealed that the maximum removal efficiency of 102.2 mg/L of the dye is about 99.67% under pH 6.0, the optimal contact time is 5 min, and the adsorbent dosage is 0.5 g/L. At 29°C; the adsorption capacity increased from 81.29 to 332.60 mg/g when the initial concentration of the dye was increased from 40.97 to 212.20 mg/L. Adsorption kinetics fitted well with the pseudo–second-order model with a good correlation (*R* ^2^ = 0.9998). The Langmuir isotherm model can best describe the adsorption isotherm model. Based on the experimental results, the CM-βCD:nChi:Glu terpolymer has a promising potential as an efficient novel adsorbent for the removal of textile dye acid red 37 from contaminated water. This study’s preparation techniques and demonstrated mechanisms offer valuable insights into the adsorbent–adsorbate interactions mechanism, analysis, challenges, and future directions of beta-cyclodextrin/chitosan–based adsorbents in wastewater treatment.

## 1 Introduction

Water is one of the most valuable reserves among all natural resources that have economic, social, political, and environmental importance worldwide (Shin et al., 2020; Ibrahim et al., 2022). Recently, water pollution has triggered significant concerns due to its adverse environmental impact. As a result of rapid industrialization and the fastest modernization, numerous pollutants are introduced into the aquatic ecosystem (Kaur and Jindal, 2019). Thus, human activities play an ever-greater part in increasing water incompetence by polluting natural water supplies. Among various water pollutants, dyes are considered the most common contaminants that have the most hazardous chemical classes. Globally, about 0.8 million tons of dyes are produced annually, making them one of the greatest important groups of industrial chemicals (Pandey et al., 2020). Synthetic dyes are produced by many industries, especially textile industries, which are the main contributors to water pollution. Moreover, many synthetic dyes are lost during the dyeing process and released into the textile effluents (Subramani and Thinakaran, 2017; Crini et al., 2019). Consequently, massive dye production, along with large-scale applications, creates significant environmental contamination, causes serious health risks, exacerbates the water crisis, and negatively influences plant and animal lives, human health, and the entire living ecosystem (Altaher et al., 2014). Because of this expanding environmental issue, color removal from sewage has received much attention. As a result, with the basic concern for protecting and conserving natural water resources, one of the major difficulties confronting the scientific community is the control of water contamination. Several physicochemical and biological techniques have been developed and have gained great attention in removing dyes from wastewater, which may or may not be safe, robust, low-cost, or capable of tackling persistent organic pollutants of low concentrations (Katheresan et al., 2018; Hynes et al., 2020; Dassanayake et al., 2021; Senousy et al., 2022). Among these techniques, the adsorption process has gained considerable attention due to its remarkable efficiency, innovation, operational simplicity, and economic and non-destructive technique (Crini et al., 2018). The critical challenge in applying the adsorption method for dye elimination is in finding a fast adsorption rate and high-adsorption-capacity adsorbent. Hence, the necessary evidence that controls the efficiency of adsorption includes the attraction force between the adsorbent and adsorbate, the active sites on the adsorbent surface, low-cost adsorbents, concentration and particle size of the adsorbent, temperature, pH, and contact time. Here comes the role of choosing a suitable adsorbent that can achieve economic and effective conditions (Pourhakkak et al., 2021; Zaimee et al., 2021). β-Cyclodextrin (β-CD) is a cyclic oligosaccharide made up of seven α-D-glucose units linked through α-(1,4) linkages. Their basket-like structure contains a hydrophobic polar chamber lined on both sides with hydroxyl groups on the inside and outside of the cavity (Badruddoza et al., 2011). β-Cyclodextrin (β-CD) has an interesting ability to create attachment complexes with a wide variety of organic and inorganic compounds through its hydrophobic cavity and hydroxyl groups in solution through host–guest interactions (Li et al., 2018; Tang et al., 2018). It is an ecofriendly and biodegradable polysaccharide that has gained significant care in environmental cleaning (Sikder et al., 2019; Blach et al., 2008; Yadav et al.). The high concentration of hydroxyl groups shows high activity and can be easily functionalized. Even though dyes can be removed, the only adsorption sites are CD cavities, resulting in a long adsorption process and equilibrium time (Yuan et al., 2017). Therefore, carboxymethyl β-cyclodextrin (CM-β-CD) has been developed to increase the adsorption capacity of β-CD by grafting it with active functional groups, such as the carboxyl groups, which may serve as highly active adsorption sites. The high adsorption efficiency of CM-β-CD can be gained by increasing the number of adsorption sites thereby being considered a potential and highly efficient adsorbent for the removal of dyes from wastewater (Pu et al., 2020; Ghazimokri et al., 2022). Chitosan (Chi) is a natural biopolymer produced by the deacetylation of chitin of the shells of crabs and shrimps. Chi is effectively renewable, highly available, biodegradable, and biocompatible and does not generate toxic products (Azlan et al., 2009; Wu et al., 2010; Santos et al., 2019). Thus, Chi as the adsorbent is suitable for removing anionic dyes. Furthermore, different physical and chemical modifications have been made to its molecular structure by the addition of crosslinking agents. For example, crosslinking chitosan with crosslinking agents such as glutaraldehyde (Glu) allows for better physical and chemical properties and improves the adsorption capacity that maximizes the removal efficiency (Filipkowska, 2012; Galan et al., 2021). Some studies have used glutaraldehyde as the flexible grafting and crosslinking agent for the reaction between chitosan and β-CD or carboxymethyl β-cyclodextrin (CM-βCD) in the preparation of two- and three-component polymers. Thus, the resulting terpolymer gains a range of sorption-based applications (Wilson et al., 2013).

The innovative aspect of this research is the development of a novel terpolymer (three-component) adsorbent material by crosslinking CM-βCD and nanochitosan with glutaraldehyde (CM-β-CD:nChi:Glu), to employ it for the removal of toxic textile dye pollutants from aqueous systems. Several tools were utilized to fully characterize the produced material, such as FTIR, XRD, SEM, TEM, BET–specific surface area (BET-SSA), and zeta potential. Then, by applying the product as a potential adsorbent material, a textile dye model to remove acid red 37 dye from the aqueous medium was developed. To our knowledge, the simultaneous removal of acid red 37 from water using such a terpolymer adsorbent has not been reported in the available literature. The adsorption behavior, mechanism, characteristics, and thermodynamics of CM-βCD:nChi:Glu have also been evaluated in this study.

## 2 Materials and methods

### 2.1 Reagents and chemicals

Materials, chemicals, and their sources are described in the Supplementary Material.

### 2.2 Methods

#### 2.2.1 Synthesis of carboxymethyl β-cyclodextrin

CM-βCD was synthesized according to the reported steps with some essential modifications (Mohamadi Zahedi and Mansourpanah, 2018; Emara et al., 2020). First, 5.0 g of β-cyclodextrin was dissolved in 50.0 mL of 10% (w/v) NaOH by constant stirring for complete mixing. After that, 10.0 mL of epichlorohydrin was added dropwise with vigorous stirring for 8.0 h. Then, 5.0 mL of epichlorohydrin was added more, and the mixture was continuously stirred for another 3 h and left overnight at room temperature. Then, the solution was concentrated to a volume of about 15.0 mL and cleansed with cold ethanol while the mixture was vigorously stirred to obtain a gummy precipitate. Washing again with cold acetone produced a durable precipitate. Thus, an excellent yield of β-CD/epichlorohydrin copolymer (12 g) was obtained. To gain our target polymer, 300 mL of 5% (W/V) NaOH solution was used to dissolve 12 g of the β-CD/epichlorohydrin copolymer, and then, 12 g of monochloroacetic acid was added gently. The mixture was vigorously stirred for 24 h. Excess NaOH was neutralized by adding 2 M HCl dropwise until neutral pH. The mixture was cooled to 4°C after being concentrated to 45 mL using an ice bath. It was filtered to remove the precipitated NaCl. Hence, the supernatant was precipitated by stirring vigorously with cold ethanol. Finally, a highly pure yield of CM-βCD was obtained by washing several times with ethanol.

#### 2.2.2 Synthesis of nanochitosan

Nanochitosan was prepared as in the literature (Taghizadeh et al., 2019) with some modifications. 2.0 g of chitosan was dissolved in 200 mL of 25% aqueous acetic acid solution and stirred for 24 h at room temperature till the solution became clear (solution A). 1.0 g of trisodium citrate was dissolved in 100.0 mL of deionized water (solution B). Solution B was gradually added to solution A, with constant stirring till a pale yellow nanochitosan solution was formed.

#### 2.2.3 Synthesis of carboxymethyl β-cyclodextrin–nanochitosan–glutaraldehyde terpolymer

CM-CD polymer (0.7506 g) was dissolved in 35.0 mL of 8.6 × 10^−2^ M acetic acid, then 25.0 mL of nanochitosan solution was added, and the mixture was stirred overnight. The mixture turned into gel by rapid addition of 25.12 mL of 25% (w/v) glutaraldehyde. After 1.5 h of continuous stirring, the temperature was raised to 60°C and the mixture was continuously stirred for another 2 h to complete the reaction. A dark orange-yellow hydrogel was obtained that was washed several times with deionized water and finally dried after about 2 h at 50°C (Wilson et al., 2013).

#### 2.2.4 Batch adsorption method for removal of acid red 37 dye from aqueous medium

Batch adsorption experiments were followed to study the adsorption behavior of the synthesized composite (CM-βCD:nChi:Glu) toward acid red 37 dye in terms of i) pH, ii) contact time, iii) initial dye concentration, iv) the adsorbent dosage, and v) temperature. Each parameter was investigated at least thrice to ensure consistent results. The concentrations of the remaining pollutant (acid red 37 dye) were determined by measuring the dye absorbance before and after the adsorption process, using a UV-Vis spectrophotometer at a wavelength of 504 nm. 250 mg/L of acid red 37 dye was prepared as the stock solution, and different concentrations were obtained by diluting in deionized water.

The adsorption capacity of acid red 37 dye on the adsorbent **(**CM-βCD:nChi:Glu**)** was determined using Equation 1 (Mittal et al., 2009)

$$q_{e}=\frac{\left(C_{0}-C_{e}\right)\;V}{m}$$ (1)

where $q_{e}$ is the amount of dye (mg) that is adsorbed by 1.0 g of the adsorbent, $C_{O}$ and $C_{e}$ are the concentrations (mg/L) of the dye at zero time and equilibrium, respectively. V is the volume of dye solution in a liter, and m is the mass (g) of the adsorbent.

The batch experiments were done at 29°C at a shaking rate of 250 rpm to study the following effects:

(i) pH: the effect of pH was studied in the range of 2.0–9.3, applying 25.0 mg of CM-βCD:nChi:Glu to remove 102.0 mg/L of the polluting dye in 50 mL solution. 1.0 M hydrochloric acid and 1.0 M sodium hydroxide solution were used to adjust the pH using a pH meter for 10 min;
(ii) contact time: appropriate time intervals in the range of 20–600 s at the optimum pH of 6.0; the amount of acid red 37 dye was 50.0 mL of 102.0 mg/L and that of CM-βCD:nChi:Glu was 25.0 mg;
(iii) initial dye concentrations, $C_{O}$ : this was studied varying from 80.30 to 214.72 mg/L at pH 6, with 25.0 mg of the adsorbent; and
(iv) adsorbent dosage: this was studied in the range 8.0–50.0 mg.

Furthermore, the effect of temperature on the adsorption process was studied in the range of 293–313 K under the optimum pH of 6.0, 50.0 mL of the dye solution (102.21 mg/L), and 25.0 mg of the adsorbent.

The removal percentage (% R) was calculated using Equation 2:

$$\%R=\frac{C_{0}-C_{e}}{C_{0}}\times 100$$ (2)

where $C_{O}$ and $C_{e}$ are the initial and equilibrium dye concentrations (mg/L).

To determine the point of zero charge (pH PZC), eight 125-mL stoppered glass bottles containing 0.1 N NaCl were prepared and adjusted to different pH values (2.0–9.3) using 0.1 N of either HCl and/or NaOH. 25.0 mg of the adsorbent (CM-βCD:nChi:Glu) was added to every bottle and the mixtures were shaken for 48 h, at room temperature, at 250 rpm shaking rate. The calculated ΔpH $\left(=pH_{after\;48h}-pH_{0}\right)$ was plotted *versus* the starting pH (pH_0_). The pH PZC was then derived from the intersection of the resulting plot with the pH_0_ axis.

The desorption experiments were examined using 50.0 mL of 1 M NaCl prepared in 60% ethanol as the eluent solution (Xing et al., 2012) and applied to the adsorbent carrying the acid red 37 dye. The adsorbent was then washed with deionized water, and the process was repeated several times. The resulting solution was examined spectroscopically every time. The recycling experiment was repeated five times.

### 2.3 Instrumentation

A double-beam UV-Vis spectrophotometer (T80+ UV/vis spectrometer, PG Instruments Ltd, UK) was used to measure the concentration of the dye at *λ* = 504 nm, while FT-IR spectra (FT-IR, KBr pellets; 3 mm thickness) were validated on a Perkin-Elmer FT-IR spectrophotometer (FT-IR 1650). Energy dispersive X-ray spectroscopy (EDX) FEI (QUANTA-250) was utilized to identify the chemical composition of the synthesized terpolymer. X-ray diffraction (XRD) analysis was used to determine the crystalline structure by (XRD-7000, Shimadzu, Japan) Cu kα radiation (*λ* = 1.54060 Å). The surface morphology was examined using a scanning electron microscope JEOL JSM-5300LV. The specific surface area of the adsorbents was calculated with the Brunauer–Emmett–Teller (BET) equation using a BELSORP-mini X (S/N: 149, Version 1.0.9.0). The zeta potential (ζ-potential) measurements of the diluted samples were determined using a Zetasizer (NANO, µV, APS) software v7.13.

## 3 Results and discussion

### 3.1 Characterization of synthesized terpolymer nano-adsorbent CM-βCD:nChi:Glu

**FIGURE 1:**
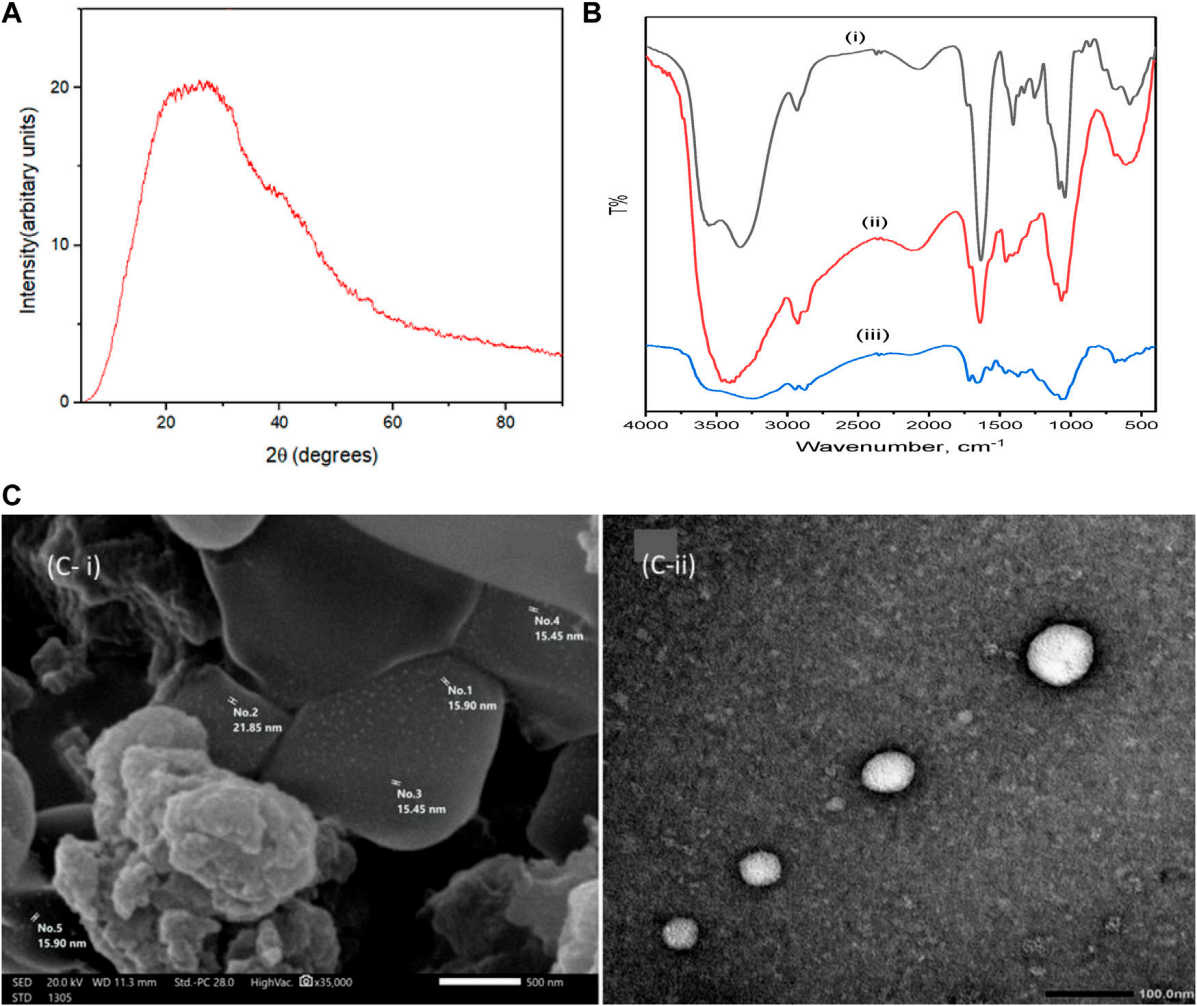
X-ray diffraction pattern of (CM-βCD:nChi:Glu), **(A)**, FTIR spectra of CM-βCD **(B i)**, CM-βCD:nChi:Glu **(B ii)**, and CM-βCD:nChi:Glu- after adsorption **(B iii)**, SEM image **(C i)** and TEM image of CM-βCD:nChi:Glu **(C ii)**.

The XRD spectrum of the amorphous structure of the nanocomposite CM-βCD:nChi:Glu is shown in Figure 1i. The spectral pattern reflects the amorphous nature of the cross-linked terpolymer (Mohamadi Zahedi and Mansourpanah, 2018; Yang et al., 2020).

The FTIR spectra of CM-βCD and CM-βCD:nChi:Glu terpolymer in the 400–4,000 cm^−1^ wave number range are shown in Figure 1ii. The spectrum of CM-βCD exhibits bands at 3,330; 2,930; 1,634; and 1,405 cm^−1^ attributed to ν_OH_, ν_CH_, ν_asy__C=O_, and ν_sy__C=O_, respectively. The bands at 1,060 and 1,040 cm^−1^ are attributed to ν C–O. The spectrum of CM-βCD:nChi:Glu terpolymer shows a broadening in 3,100–3,550 cm^−1^ that may be attributed to the cross-linkage bonding between nanochitosan and CM-βCD. The broad band appearing at 2,927 cm^−1^ corresponds to the νCH that overlaps with the OH stretching peak (Chen and Park, 2003). The bonding between the carboxylic group of CM-βCD and the amino group of chitosan is evidenced in the shifts from 1,639 cm^−1^ and 1,457 cm^−1^ to 1,633 cm^−1^ and 1,405 cm^−1^, respectively (Wang et al., 2016).

The surface morphology of CM-βCD:nChi:Glu is determined by scanning electron microscopy, showing a heterogeneous surface due to the non-uniform cross-linking interactions. Figure 1Aiii shows an irregular surface structure with holes and high cavities increasing the surface area and can be the reason for the effective adsorption properties (Pu et al., 2020). The TEM image in Figure 1Biii shows the highly stable and well-dispersed spherical CM-βCD:nChi:Glu nanoparticles in the size range 30–72 nm.

CM-βCD:nChi:Glu was exposed to BET technique to determine the surface area and pore distribution *via* a nitrogen adsorption–desorption method. As can be predicted, this terpolymer shows the low specific surface area (2.903 m^2^ g^−1^), poor pore volume (0.002 cm^3^ g^−1^), and average pore diameter (3.078 nm).

**FIGURE 2:**
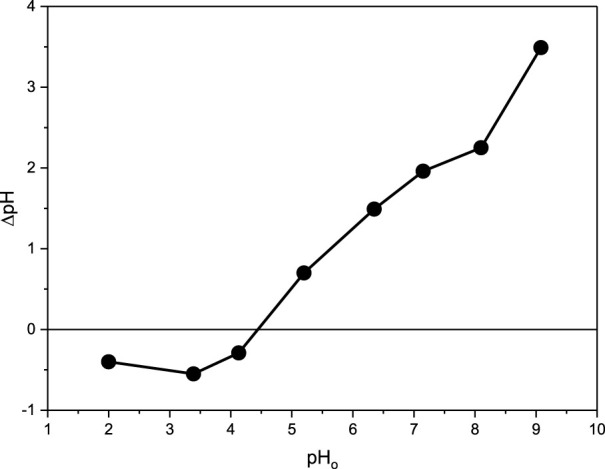
Point of zero charge of CM-βCD:nChi:Glu.

The surface charge density of the adsorbent is explained based on the point of zero charges, at which the charge on the adsorbent surface is zero (Behbahani et al., 2020; Elbadawy et al., 2021). Plotting the relationship between ΔpH and pH_0_, the point of zero charges was found to be 4.5 after 48 h (Figure 2). Thus, a positive charge was formed on the CM-βCD:nChi:Glu surface when the solution pH was less than the calculated zero-point charge value (4.5), while a negative charge was developed when the pH was more than 4.5. The free negative charge on the CM-CD:nChi:Glu attracts the dye, promoting the adsorption process (Islam et al., 2019). Furthermore, the zeta potential was measured and showed a surface potential of 20.8 mV, confirming the positivity of the adsorbent’s surface. Also, this high positive charge reflects the electrical stability of CM-CD:nChi:Glu because the surface charge inhibits particle aggregation and becomes coagulation-resistant.

### 3.2 Factors affecting adsorption behavior

The adsorption behavior of the synthesized nano-composite CM-βCD:nChi:Glu toward acid red 37 dye was studied using batch adsorption techniques. The main parameters studied are i) pH, ii) contact time, iii) initial dye concentration, and iv) adsorbent dosage.

#### 3.2.1 pH Effect

**FIGURE 3:**
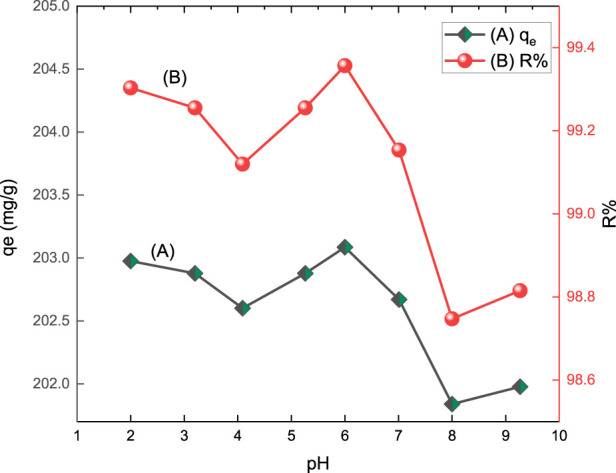
Effect of pH on **(A)** adsorption capacity (q_e_) and **(B)** % removal (R%) of acid red 37 dye by CM-βCD:nChi:Glu.

The pH of a solution is one of the essential variables affecting the adsorption process related to the adsorbate and surface characteristics of the adsorbents. The acid red 37 dye removal by CM-βCD:nChi:Glu adsorbent was explored in the pH range of 2.0–10.0 by shaking 25 mg of adsorbent with 102.2 mg L^−1^ of the dye solution for 10 min at 250 rpm. The % removal of the dye and the adsorption capacity onto CM-βCD:nChi:Glu as a function of the pH are presented in Figure 3. The highest adsorption efficiency was found at pH 6.0. Some variations in the adsorption efficiency were noticed in the applied pH range (Wu et al., 2018). This can be attributed to the pH impact of the medium on the surface charge of the adsorbent. As deduced from the zeta potential and PZC tests, the charge on the surface of the adsorbent is positive, which attracts the negatively charged dye at pH 2.0, showing relatively high adsorption of 99.3%, then a decrease is noticed till pH 4.5, where the adsorbent charge becomes zero after which its surface becomes negative, attracting the protonated dye till pH 6.0, with the highest efficiency of 99.8%. Increasing the pH of the medium stabilizes the dye and adsorbent as negatively charged species, decreasing the adsorption efficiency. Previous reports on the adsorption of anionic dyes such as methyl orange or Congo red using chi- and βCD-based adsorbents, separately or mixed, have shown that the pH of the medium has different impacts, with a higher capacity at low to moderate pH values but lower adsorption at higher pH (Ozmen and Yilmaz, 2007; Jiang et al., 2018; Jiang et al., 2020) which is consistent with the behavior of βCM-CD:nChi:Glu toward acid red 37 textile dye. However, the adsorption efficiency ranges from 98.75 to 99.36% all over the pH range of 2.0–9.3.

#### 3.2.2 Contact time effect

**FIGURE 4:**
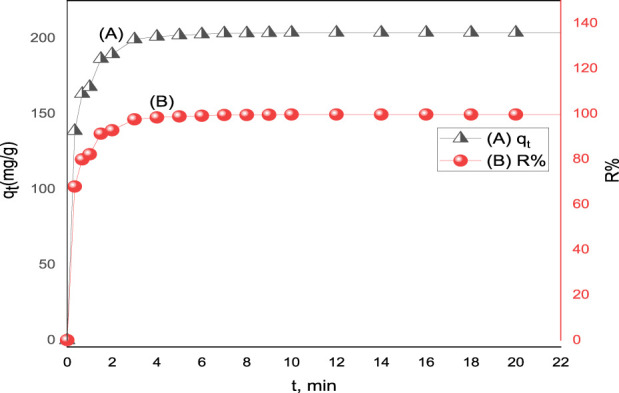
Effect of contact time on **(A)** adsorption capacity (q_t_) and **(B)** % removal (R%) of acid red 37 dye on CM-βCD:nChi:Glu (25 mg of adsorbent with dye initial concentration C_o_ = 102.2 mg L^−1^, the optimum pH, and shaking rate of 250 rpm).

The adsorption of acid red 37 dye by the synthesized adsorbent CM-βCD:nChi:Glu was investigated at different adsorption times from 20 s to 10 min. The effect of the contact time on dye adsorption onto the prepared adsorbents is displayed in Figure 4. The results indicated that the effectiveness of dye removal increases with increasing contact time until an equilibrium is achieved. An instantaneous increase in the adsorption capacity is observed up to 5.0 min, and then it slowly increases to become almost constant at 10 min with a removal efficiency of 99.67% (Jawad et al., 2021). The rapid adsorption at the beginning can be explained on the basis of the vacant active adsorption sites on CM-βCD:nChi:Glu, but eventually this slows down with time and remains almost the same. The optimum time of contact for the adsorbents was 3–5 min (Islam et al., 2019).

#### 3.2.3 Initial dye concentration effect

**FIGURE 5:**
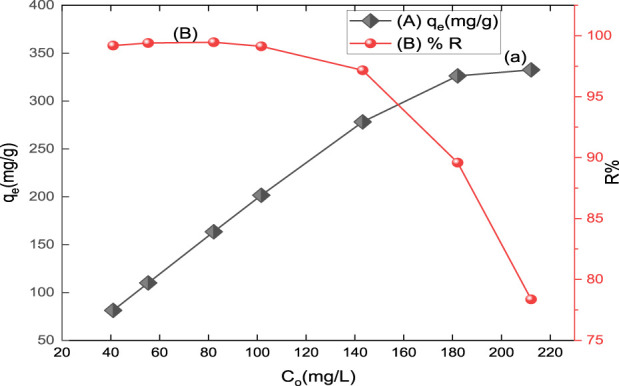
Effect of initial concentration on **(A)** adsorption capacity (q_t_) and **(B)** % removal (R%) of acid red 37 dye on CM-βCD:nChi:Glu (25 mg of the adsorbent for 5.0 min and pH 6.0).

The dye adsorption by CM-βCD:nChi:Glu has been carried out as a function of the initial concentration of the dye from 40.97 mg/L to 212.20 mg/L (Figure 5). Increasing the initial dye concentration led to a greater adsorption capacity for CM-βCD:nChi:Glu, but a lower percentage of removal. If more dye was present at the onset, more binding sites would become occupied, resulting in a higher adsorption capacity. It is also likely that the initial high concentration of the adsorbate molecules will result in a more direct contact between the dye and adsorbent particles. There exists the possibility that an insufficiency in the number of available active sites on the adsorbent surface could be responsible for the decrease in dye removal that occurs when the initial dye concentration is increased (Si et al., 2015).

#### 3.2.4 Adsorbent dosage effect

**FIGURE 6:**
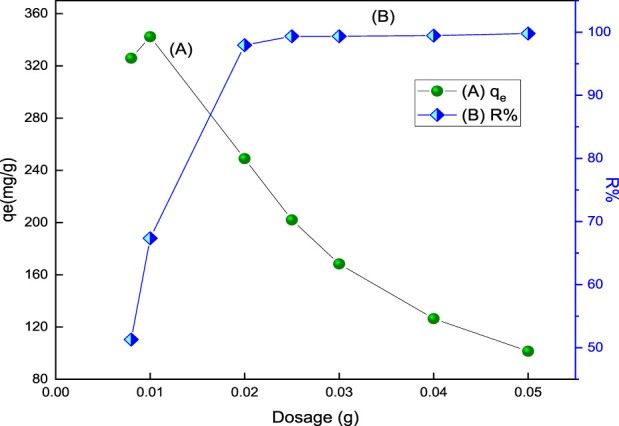
Effect of adsorbent, CM-βCD:nChi:Glu, dosage on **(A)** the adsorption capacity and **(B)** % removal (R%) of acid red 37 (102.2 mg L^−1^ dye solution for 30 min at 250 rpm and optimum pH).

The % removal of acid red 37 dye and its adsorption capacity onto the prepared adsorbent as a function of the adsorbent dosage varying from 8.0 mg to 50.0 mg are shown in Figure 6. The result reveals that the percentage removal increases as the mass of the adsorbent increases till the optimum dosage. The optimum dosage that can be used for the adsorption process is 25 mg for CM-βCD:nChi:Glu. On the other hand, the adsorption capacity decreases relative to the dosage of the adsorbent. The increase in adsorption removal efficiency with increasing dosage of the adsorbents is expected due to the availability of additional surface area in the adsorption process. Moreover, no significant increment in adsorption is observed after the addition of a certain amount of the adsorbent, and this point is known as the optimum dose (25 mg). This is because of the equilibrium condition as the number of dye anions attached to the surface of the adsorbent and the number of mobile anions remains constant. Hence, any further addition of the adsorbent does not change adsorption significantly (Abdulhameed et al., 2019).

### 3.3 Adsorption kinetics

The adsorption kinetics of acid red 37 dye onto the surface of the synthesized adsorbent CM-βCD:nChi:Glu was investigated at a pH of 6.0°C and temperature of 29°C, applying the pseudo–first- and second-order, Elovich, and intraparticle diffusion kinetic models (Ho and Mckay, 1999; Yuh-Shan, 2004; Priscila et al., 2013; Wang et al., 2019). Supplementary Table S1 summarizes the examined adsorption kinetic models in Supplementary Equations S1–S4.

**FIGURE 7:**
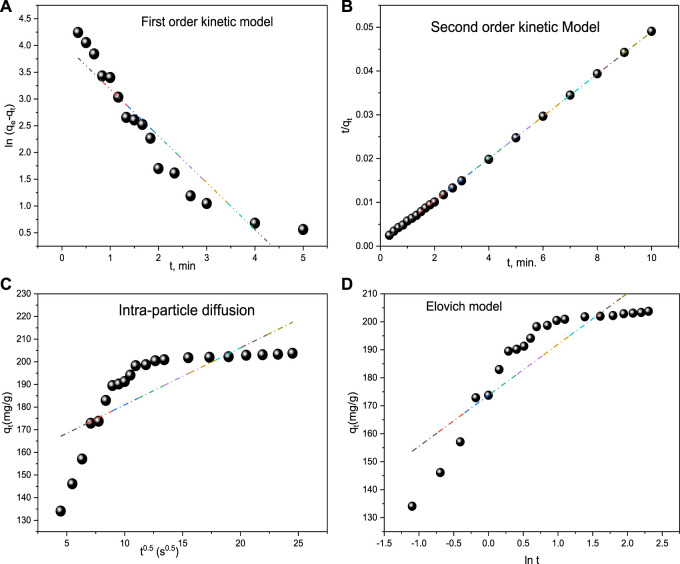
Kinetic models **(A)** First order kinetic model, **(B)** Second order kinetic model, **(C)** Intra-particle diffusion and **(D)** Elovich model for acid red 37 dye adsorption onto CM-βCD:nChi:Glu.

To determine whether the type of adsorption is physio-sorption or chemisorption, the pseudo–first-order and second-order kinetic models are applied. Figures 7A, B shows the experimentally determined values of rate constants K_1_ and K_2_ obtained from plotting the two models. The second-order model appears to be more favorable for the dye adsorption process, indicating its chemical adsorption by CM-βCD:nChi:Glu (Eltaweil et al., 2020). The $K_{1}$ and $K_{2}$ values for CM-βCD:nChi:Glu and the other parameters obtained from the linear form of pseudo–first-order and second-order are listed in Table 1.

**TABLE 1:** Kinetic parameters of the sorption of acid red 37 dye by CM-βCD:nChi:Glu.

Kinetic model	Parameters		Equation
Pseudo–first order	q_e_ (mg g^−1^) (Exp./Calc.)	203.76/57.92	y = −0.8745x + 4.0592
	k_1_ (min^−1^)	0.876	R^2^ = 0.8852
Pseudo–second order	q_e_ (mg g^−1^) (Exp./Calc.)	203.76/208.33	y = 0.0048x + 0.0007
	k_2_ (g mg^−1^ min^−1^)	3.2 × 10^−2^	R^2^ = 0.9998
Elovich	β (g mg^−1^)	0.0549	y = 18.221x + 173.74
	α(mg g^−1^ min^−1^)	2.5 × 10^5^	R^2^ = 0.775
Intra-particle diffusion	C_i_ (mg g^−1^)	155.84	y = 2.5146x + 155.84
	k_id_ (mg g^−1^ min^−0.5^)	19.48	R^2^ = 0.5715

C_i_; External film resistance.

In addition, the Elovich kinetic model is examined to identify if the adsorption mechanism is chemisorption. The chemisorption mechanism usually involves energetically heterogeneous adsorbent surfaces. The Elovich constants (initial adsorption rate, α: 115.9 mg g^−1^ min^−1^ and the surface coverage and activated energy, β: 0.0201 g mg^−1^) give an insight into the energetic heterogeneity of the surface (Figure 7C).

Furthermore, the adsorption of the targeted material onto the active sites of the adsorbent, mass transfer across the liquid film surrounding the surface, and intra-particle diffusion onto the pores of the surface make up the three stages of the intra-particle diffusion model. The processes that make use of the active sites in the adsorbents are thought to go faster. Now, if the model fits, the plot of q_t_ *versus* t^0.5^ should show a straight line, either i) passing through the origin, when the intra-particle diffusion is the only controlling step or ii) with an intercept with the *y*-axis (Figure 7C) (Özer et al., 2006; Singh et al., 2012; Haerifar and Azizian, 2013). However, after fitting the data to the intra-particle diffusion model, a multi-linear plot was seen, indicating that intra-particle diffusion and bulk-to-surface transit of acid red 37 dye interact together producing the observed response. Intra-particle diffusion is not the rate-limiting phase since the plot does not pass through the origin. k_id_ and C_i_ were found to be 2.514 mg g^−1^ s^−0.5^ and 155 mg g^−1^, respectively.

### 3.4 Adsorption equilibrium isotherm models

**FIGURE 8:**
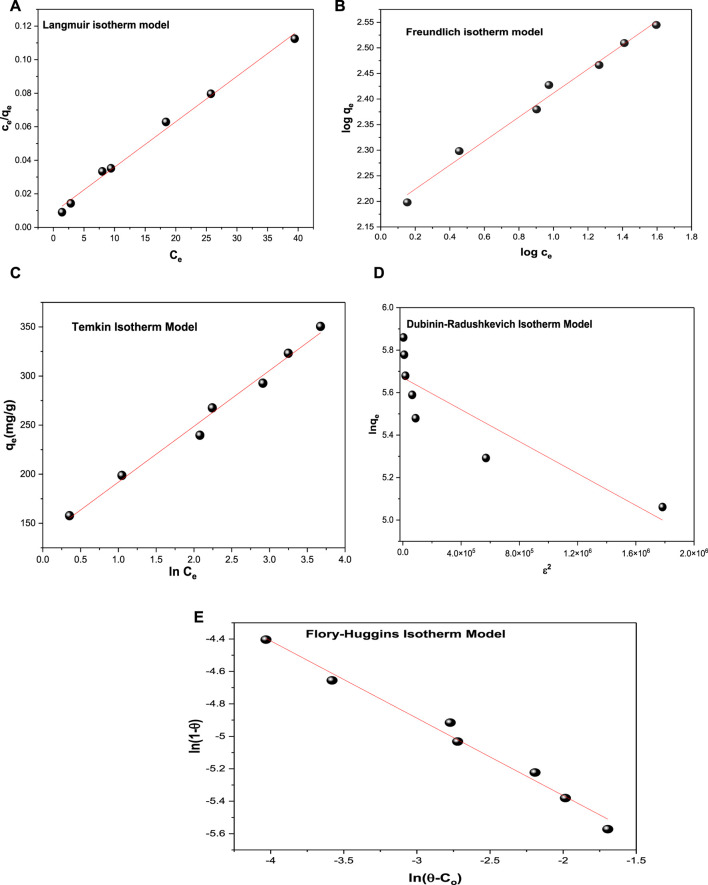
Isotherm models **(A)** Langmuir isotherm model, **(B)** Freundlich isotherm model, **(C)** Temkin isotherm model, **(D)** Dubinin–Radushkevich isotherm model and **(E)** Flory–Huggins isotherm model for dye adsorption onto CM-βCD:nChi:Glu.

The adsorption isotherm models are applied to relate the equilibrium and adsorbed concentrations of acid red 37 dye (Figure 8) and thus estimate the adsorption efficiency of the adsorbent CM-βCD:nChi:Glu. The linear equations of the applied isotherm models are given in Supplementary Table S2, expressed as Supplementary Equations S5–S9.

The values of the correlation coefficients *R* ^2^ of the linear plots of the applied models are typically compared for the evaluation of the best fit (Table 2). The *R* ^2^ values can be arranged as: Langmuir > Freundlich> Temkin > Flory–Huggins > Dubinin–Radushkevich.

**TABLE 2:** Adsorption isotherm parameters of the sorption of acid red 37 dye by CM-βCD:nChi:Glu.

Isotherm model	Parameters		Equation
Langmuir	q_m_ (mg g^−1^)	370.37	y = 0.0027x + 0.0088
	K_L_ (Lmg^−1^)	0.3068	R^2^ = 0.9933
	R_L_	(0.0390–0.0149)	—
Freundlich	1/n	0.2345	y = 0.2345x + 2.1771
	K_F_	150.349	R^2^ = 0.987
Temkin	ß_T_ (J mol^−1^)	44.608	y = 56.846x + 135.11
	K_T_ (L g^−1^)	10.770	R^2^ = 0.9874
Dubinin–Radushkevich	q_m_ (mg g^−1^)	290.122	y = −4.0 × 10^−7^x + 5.6703
	ß_DR_ (mol^2^/J^2^)	4.0 × 10^−7^	R^2^ = 0.773
	E (J mol^−1^)	1,118.034	—
Flory–Huggins	n_FH_	−0.4766	y = −0.4766x − 6.3182
	k_FH_ (Lmol^−1^)	0.0018	R^2^ = 0.9848
	ΔG°(Jmol^−1^)	−15863.91	—

From the Langmuir isotherm model, the values of the maximum adsorption capacity (q_m)_ and Langmuir isotherm constant (K_L)_ were calculated to be 370.37 mg g^−1^ and 0.3068 L mg^−1^, respectively. The equilibrium parameter R_L_ = (0.0390–0.0149) values postulate an advantageous adsorption behavior of the CM-βCD:nChi:Glu adsorbent toward the acid red 37 dye, leading to the assumption that sorption sites are energetically equivalent, and the acid dye removal occurs on a homogeneous surface by monolayer adsorption. The Freundlich model was also applied, and the experimental results revealed a linear association between log q_e_ and log C_e_; the adsorption intensity (1/n) = 0.2345 < 1 (*n* = 4.264) and adsorption capacity (K_f_) = 150.35 mg/g.

In order to examine if the adsorption possesses a uniform bond energy distribution, the Temkin isotherm model was applied (Tempkin and Pyzhev, 1940). With values of the Temkin isotherm constant (K_Te_) and Temkin constant related to the heat of sorption (b_Te_) being 10.77 L g^−1^ and 44.61 J mol^−1^, respectively, the Temkin isotherm fits the adsorption process well.

On the other hand, Dubinin–Radushkevich isotherm shows *R* ^2^ is equal to 0.773, which is less fitting with the experimental data. While the Flory–Huggins model (Supplementary Equation S11) is better fitting with *R* ^2^ equal to 0.9848, with the Flory–Huggins constants (n_FH_, k_FH_) being equal to −0.4766 and 0.0643 Lmol^−1^, respectively, and the ΔG° value being −15863.91 Jmol^−1^. The Flory–Huggins isotherm describes the adsorbate’s surface coverage patterns on the adsorbent.

### 3.5 Thermodynamics of acid red dye adsorption

The thermodynamic parameters such as the change in free energy ∆G^o^, kJ/mole; change in enthalpy ∆H^o^, kJ/mole; and change in entropy ∆S^o^, kJ/mole/K) provide valued information about the type and mechanism of the adsorption process. The change in Gibbs free energy (∆G^o^) of an adsorption process denotes its spontaneity. A negative ∆G^o^ value indicates spontaneous adsorption. Equations applied to deduce the thermodynamic parameters are given in Supplementary Table S3, expressed as Supplementary Equations S10–S12 (Dizge et al., 2008; Tanhaei et al., 2019).

**FIGURE 9:**
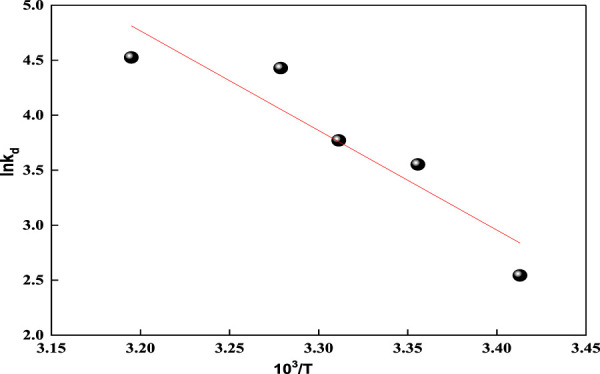
Representation of Van't Hoff equation for acid red 37 dye adsorption onto CM-βCD:nChi:Glu.

By applying the Van 't Hoff equation (Supplementary Equation S13) in plotting ln K_d_ against 1/T as shown in Figure 9, a straight line is obtained with a slope equal to ΔH^o^/R and an intercept of ΔS^o^/R (Anush and Vishalakshi, 2019). Table 3 presents the thermodynamic parameters for acid red 37 dye adsorption onto the CM-βCD:nChi:Glu adsorbent studied at the temperature range 293–313 K. The negative values of ΔG° indicate the spontaneous nature of the adsorption processes, which are adjusted by chemical adsorption, whereas decreases in ΔG° with rising temperatures show more favorable adsorption at higher temperatures. Also, the positive values of ΔH^o^ and ΔS^o^ reflect endothermic adsorption with additional randomness states existing at the solid–liquid interfaces.

**TABLE 3:** Thermodynamic parameters for dye adsorption onto CM-βCD:nChi:Glu.

Temperature (K)	lnK_d_	ΔG° (KJmol^−1^)	ΔH° (KJmol^−1^)	ΔS° (Jmol^−1^K^−1^)
293	2.543	−6.195	75.289	280.556
298	3.552	−8.800		
302	3.771	−9.469		
305	4.429	−11.230		
313	4.525	−11.775		

### 3.6 Desorption and reusability study

**FIGURE 10:**
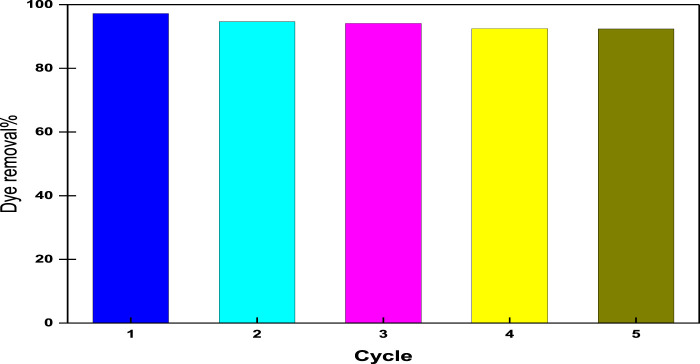
Reusability of CM-βCD:nChi:Glu for the adsorption of acid red dye 37 (dye concentration, 102.21 mg L^−1^; adsorbent dose, 0.025 g; time, 5 min; volume of dye solution, 50 mL; the optimum pH, and shaking rate, 250 rpm).

From the point of view of economical, ecological, and material science, the reusability of adsorbents is a fundamental issue. Consequently, it is desirable to adsorb pollutants and regenerate the adsorbent for many cycles. Desorption is accomplished by incubating the terpolymer adsorbent with a solution of 60% ethanol and 1 M NaCl for 20 min; the regenerated adsorbents are then washed many times with distilled water. The regenerated adsorbents were used for five successive adsorption–desorption cycles and are presented in Figure 10. It has been observed that the dye removal efficiency of CM-βCD:nChi:Glu after five cycles decreased from 97.2 to 92.4%. This decrease may be attributed to the possibility of breakage of the polymer chains as a result of repeated base treatment during the reusability process. Since the examined acid red 37 dye is of the anionic type, CM-βCD:nChi:Glu can be considered an efficient adsorbent for anionic dyes.

### 3.7 Suggested adsorption mechanism of acid red 37 dye by CM-βCD:nChi:Glu

**SCHEME 1:**
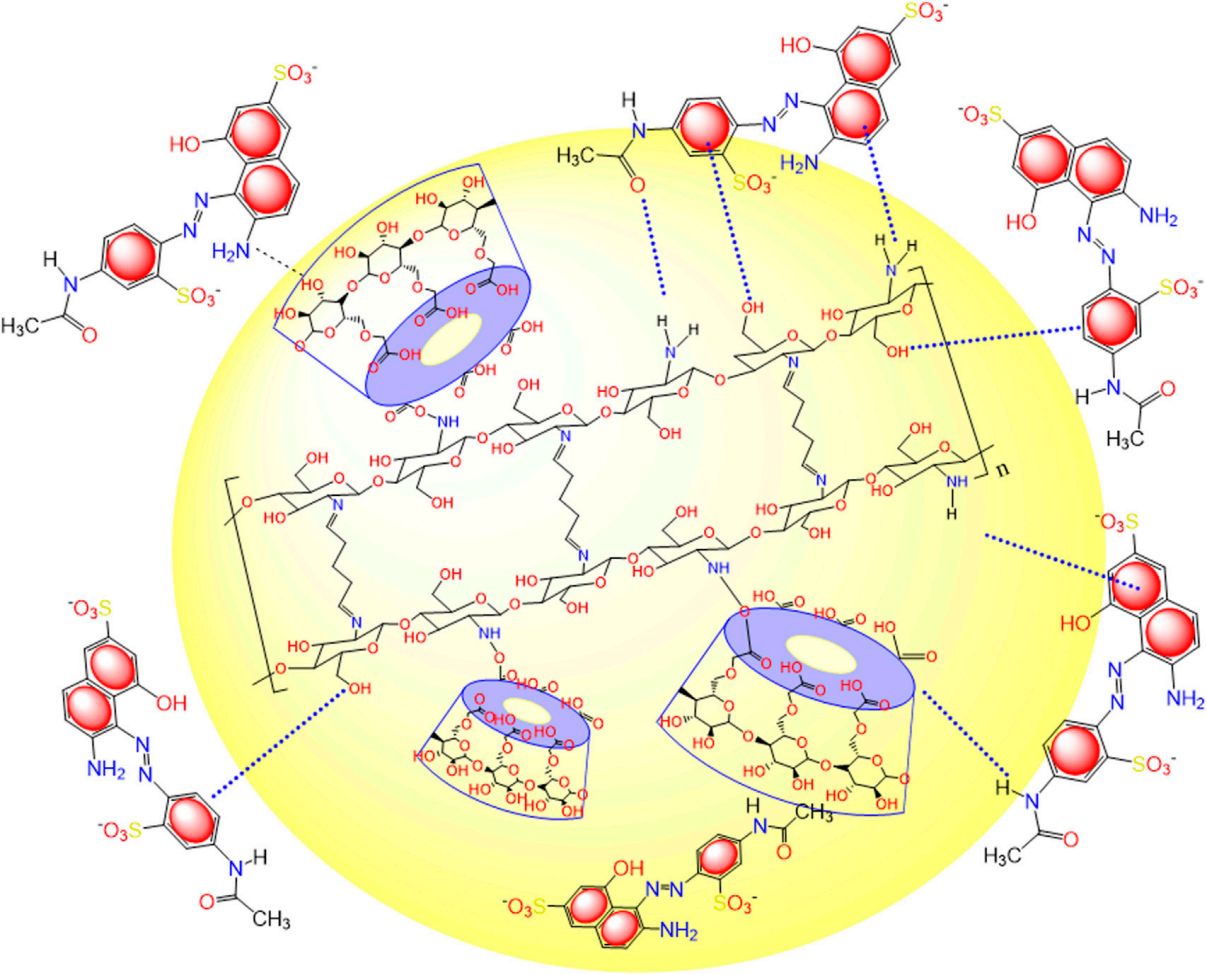
Illustration of the probable interactions between CM-βCD:nChi:Glu and acid red 37 dye.

The adsorption mechanism of acid red 37 dye on the surface of CM-βCD:nChi:Glu is displayed in Scheme 1. A comparison between the FTIR analysis of the adsorbent before and after the adsorption process shows the interaction of acid red 37 dye with CM-βCD:nChi:Glu (Figures 1B, C, ii). The adsorbent band centered at 3425 cm^−1^ corresponds to O-H and N-H stretching, showing different features with more broadening (3,000–3,600 cm^−1^), indicating their interactions with the adsorbed dye. Moreover, a peak appears at 1,554 cm^−1^ (Figure 1Cii), attributed to the vibration of the aromatic ring of the dye molecules. However, the adsorption mechanism could be explained by the synergistic effect of the electrostatic attraction of the amino groups from nanochitosan with host–guest interactions from β-cyclodextrin. Thus, cyclodextrin cavities are responsible for capturing organic pollutant molecules *via* the host–guest inclusion interaction (Zhao et al., 2017; Pu et al., 2020; Shi et al., 2022). Also, the negatively charged sulfonate groups (-SO_3_^−^) of acid red 37 dye facilitate electrostatic attractions with the amino and hydroxyl groups of chitosan. Moreover, other interactions may take place, but with lesser opportunity, such as dipole–dipole bonds, hydrogen bonding, electrostatic, Van der Waals, hydrophobic, n–pi interactions, and porous network capture (Jiang et al., 2020; Galan et al., 2021). We also found that the adsorption process followed pseudo–second-order kinetics, suggesting chemical sorption. The affinity of CM-βCD:nChi:Glu as the adsorbent for the acid red 37 dye was verified by negative values of free energy change (ΔG°). The high adsorption efficiency may be attributed to the triple adsorption effect of CM-βCD:nChi:Glu (Jiang et al., 2020).

## 4 Conclusion

Terpolymer nano-adsorbent carboxymethyl β-cyclodextrin–nanochitosan–glutaraldehyde (CM-βCD:nChi:Glu) was successfully prepared with holes and high cavities surface structure that was characterized by FTIR, XRD, SEM, TEM, BET, and zeta potential analysis. CM-βCD:nChi:Glu composite was found to be highly efficient in removing the toxic textile acid red 37 dye. Based on the results, the adsorbent dose, pH, and initial dye concentration had significant effects on dye removal. The normal pH of 6.0, adsorbent dose of 0.5 g/L, and initial dye concentration of 102.2 mg/L were found to be the optimal conditions for dye removal with an optimum contact time of only 5 min, showing a maximum removal efficiency of 99.67%. Kinetically, the dye removal fitted best with the pseudo–second-order model with a good correlation (*R* ^2^ = 0.9998). Among the applied equilibrium isothermal models, the Langmuir isotherm model showed good agreement with the experimental data, with high *R* ^2^ and maximum adsorption capacity (332.60 mg/g). The negative values of Gibbs free energy (ΔG°) calculated from the thermodynamic data indicated that the adsorption process was spontaneous in nature. The positive values of ΔH° and ΔS^o^ reflect the endothermic adsorption process with an additional randomness state. It can therefore be said that the CM-βCD:nChi:Glu terpolymer nanocomposite has high efficacy in anionic dye removal with the benefit of low cost for easier reusing or recycling for 5 cycles. The incorporation of nanochitosan in addition to the cone structure of βCD combined together by Glu gave the adsorbent splendid properties as a highly efficient adsorbent toward the acid red 37 dye which is an example of a highly toxic anionic dye.

## Data Availability

The original contributions presented in the study are included in the article/Supplementary Material; further inquiries can be directed to the corresponding author.
