# Supplementary material for: A novel terpolymer nanocomposite (carboxymethyl β-cyclodextrin–nano chitosan–glutaraldehyde) for the potential removal of a textile dye acid red 37 from water
Source: Front Chem. 2023 Feb 1;11:1115377. doi: 10.3389/fchem.2023.1115377 (PMC9929948; doi:10.3389/fchem.2023.1115377)
Supplement: Supplementary file 1 [file DataSheet1.pdf]

Supplementary materials

A novel terpolymer nanocomposite (Carboxymethyl β-cyclodextrin–nanochitosan–glutaraldehyde) for the potential removal of a textile dye acid red 37 from the water

Hemmat A Elbadawy[1*], **Ali El-Dissouky**[1†], **Seham M. Hussein**[1], **Sara R. El-Kewaey**[1], **Souad A. Elfeky**[2†], **Gamal El-Ghannam**[2]

[1]Chemistry Department, Faculty of Science, Alexandria University, Alexandria, Egypt.

[2] National Institute of Laser Enhanced Sciences (NILES), Department of laser applications in Metrology, photochemistry, and agriculture, Cairo University, 12613, Giza, Egypt

*** Correspondence:**
Hemmat A Elbadawy
Hemmatabdelfattah@alexu.edu.eg

Keywords: terpolymer, CM-βCD:nChi:Glu, acid red 37, textile dye, adsorption capacity, % removal

Materials and methods

2.1 Reagents and chemicals

β-Cyclodextrin and chitosan (High molecular weight) were purchased from ALPHA CHEMIKA. Glutaraldehyde from ADVENT CHEMBIO PVT LTD. Epichlorohydrin was purchased from LOBA Chemie, India. Monochloroacetic was purchased from acid Research-Lab Fine Chem Industries. Absolute ethanol, hydrochloric acid, acetic acid, acetone, and trisodium citrate and sodium hydroxide were of analytical grade and purchased from Sigma-Aldrich Chemical Company, Germany.

Table (S1): Examined adsorption kinetic model equations

Model	Equation	Abbreviations (units)	Equation number
**(a) First order**	$\ln (q_e - q_t) = \ln q_e - k_1 t$	q_e (mg g^{-1}); amount of dye adsorbed on adsorbent at equilibrium<br><br>q_t (mg g^{-1}) amount of dye adsorbed on adsorbent at time t<br><br>t; time (s)<br><br>K_1 (s^{-1}) is the rate constant	(S1)
**(b) Second order**	$\frac{t}{q_t} = \frac{1}{K_2 {q_e}^2} + \frac{1}{q_e} \times t$	K_2 (g mg^{-1} s^{-1}) is the rate constant of the pseudo-second-order	(S2)
**(c) Elovich kinetic model**	$q_t = (1/\beta) \ln \alpha \beta + (1/\beta) \ln t$	α ; the initial adsorption rate (mg g^{-1} s^{-1})<br><br>β; the surface coverage and activated energy (g/mg).	(S3)
**(d) Intra-particle diffusion model**	$q_t = k_{id} t^{0.5} + C_i$	k_{id}(mg g^{-1} $s^{-1/2}$) is the intra-particle diffusion rate constant.<br><br>C_i is the external film resistance which indicates the thickness of boundary layer	(S4)

Table (S2): Linear equations of isotherm models

Isotherm model	Linear Equation	Abbreviations	Equation number
**Langmuir**	$\frac{C_e}{q_e} = \frac{1}{q_m \times k_l} + \frac{C_e}{q_m}, R_L = \frac{1}{1+K_L C_o}$	C_e (mg/L); the equilibrium concentration<br>C_o (mg/L); the initial concentration of the adsorbate ions.<br>q_e (mg/g); the amount of adsorption at equilibrium<br>q_m(mg/g); maximum adsorption capacity<br>K_L(L/mg); the Langmuir isotherm constant<br>R_L; the separation factor or equilibrium parameter ($0 < R_L < 1$)	(S5)
**Freundlich**	$\log q_e = \log k_f + \frac{1}{n} \log C_e$	K_f (mg/g) is the measure of adsorption capacity<br>(1/n) is the adsorption intensity	(S6)
**Temkin**	$q_e = \frac{RT}{b_{Te}} \ln K_{Te} + \frac{RT}{b_{Te}} \ln C_e$	K_{Te} (L/g); Temkin isotherm constant.<br>b_{Te} (J/mol); Temkin constant related to heat of sorption.<br>R (8.314 $J.mol^{-1}.K^{-1}$) is the universal gas constant.<br>T (°K); the absolute temperature	(S7)
**Dubinin-Radushkevich**	: $E = 1/(2B)^{0.5}$, $\varepsilon = RT \ln(1+ 1/C_e)$ and $\ln(q_e) = \ln(q_m) – B \varepsilon^2$<br>$E = \frac{1}{\sqrt{2B}}$, $\ln q_e = \ln q_m - B\varepsilon^2$<br>$\varepsilon = RTln(1 + \frac{1}{C_e})$	E (KJ/mol); the mean free energy of adsorption<br>B; constant related to the mean free energy of adsorption per mol of adsorbate.<br>ε ;the Polanyi potential.	(S8)
**Flory-Huggins**	$K_{FH} = \exp(−\Delta G^o/RT)$,<br>$\log(\theta/C_o) = \log(k_{FH}) + n_{FH} \log(1-\theta)$ and $\theta = 1- (C_e /C_o)$<br>$\log \frac{\theta}{C_0} = \log k_{FH} + n_{FH} \log(1-\theta)$<br>$\theta = 1 - \frac{C_e}{C_o}$, $K_{FH} = \exp(\frac{-\Delta G^o}{RT})$	ΔG^o is the standard Gibbs free energy change<br>θ; the degree of surface coverage<br>K_{FH} is the Flory-Huggins equilibrium constant.<br>n_{FH} is the ions number occupying adsorption sites	(S9)

Table (S3) Thermodynamic equations

Equation		Equation number
$\Delta \boldsymbol{G}^{\text{o}} = -\boldsymbol{RT}\ \ln \boldsymbol{K_d}$		(S10)
$\boldsymbol{K_d} = -\boldsymbol{C_e}\ /\boldsymbol{C}_{\text{o}}$		(S11)
$\Delta \boldsymbol{G}^{\text{o}} = \Delta \boldsymbol{H}^{\text{o}} - \boldsymbol{T}\Delta \boldsymbol{S}^{\text{o}}$		(S12)
$\boldsymbol{ln\ K_d} = -\ (\ \Delta \boldsymbol{H}^{\text{o}}\ /\boldsymbol{R}\ )\ (\boldsymbol{1}/\ \boldsymbol{T}) + \Delta \boldsymbol{S}^{\text{o}}/\ \boldsymbol{R}$		(S13)
Abbreviations (units)	R; the universal gas constant (8.314 J $mol^{-1}K^{-1}$)<br>T; the absolute temperature (°K)<br>K_d; the distribution constant (dimensionless)<br>$C_{o;}$ the original concentration (mg L^{-1})<br>C_e; the equilibrium concentrations (mg L^{-1})<br>ΔG^o, free energy change (kJ/mole)<br>ΔH^o ,enthalpy change (kJ/mole)<br>ΔS^o , entropy change (kJ/mole/^{o}K)	
